# Health related taxes in focus: a win–win

**DOI:** 10.1093/eurpub/ckae043

**Published:** 2024-03-07

**Authors:** Pekka Puska

**Affiliations:** Finnish Institute for Health and Welfare (THL), Mannerheimintie 166, Helsinki 00300, Finland

Many Western countries struggle nowadays with the pressures of the state budget. Inflation rates, coronavirus disease 2019 costs, Ukrainian war, increasing health care costs, etc., put the governments under stress. The responses vary from budget cuts to increased taxes. Both cuts and increased taxes are politically not easy. Cuts can easily harm disadvantaged groups, and increase taxes also harm economic growth.

In this situation health experts have referred to the potential contribution of health related ‘sin taxes’, like those increasingly used for sustainable environmental and climate developments.

Tobacco excise tax has widely been used and price is generally seen as the strongest means to reduce tobacco use.^1^ Generally, European countries have responded, but the range of tax in the tobacco price varies in the EU area, from 91% in Finland to 70% in Germany, not to speak of the much lower level in Eastern Europe.^2^ Recently these taxes have also been extended to the new nicotine products in the market.

Alcohol tax is more difficult, because of the cultural and agricultural differences related to alcohol even in Europe and to the different categories of alcohol drinks. In Northern Europe alcohol is rather heavily taxed, bringing also considerable revenues to state budgets.

Since Europe is the region with highest alcohol consumption and obviously with highest related problems, the European Regional Office of WHO has repeatedly called for stronger measures, including heavier taxation, to reduce alcohol related harms.^3^

A more recent and considerable revenue possibility is unhealthy foods. Even if diet as a whole is probably the most important determinant of noncommunicable diseases, the problem is the multiple nature of unhealthy foods and consequently the criteria for health-related taxation.

The discussion has been fueled by the growing epidemic of obesity, with its big public health consequences and societal costs, bringing sugar taxing in focus. This has generally taken place in form of taxes on sugar sweetened beverages, particularly in view of their role on childhood obesity.^4^

Both the World Bank and WHO recommend this tax that in some form has been implemented in over fifty countries.^5^ The World Bank lists four benefits of such tax: (i) Higher price reduces consumption. (ii) Staged tax promotes reformulation of the products. (iii) The tax brings revenues to the government, and (iv) It is a public sign for need to reduce sugar.

As studies increasingly show the impact of sugar drink tax^6^^,^^7^ many experts and even counties have started to consider for broader public health benefits extended taxes on unhealthy foods, especially concerning other major sources of sugar and possibly also concerning products with high salt or saturated fat.

Discussions have often proposed that instead of price increases with taxes the prices of healthier products should be lowered. This would, of course, be welcome but involve considerable costs. It can also be questioned whether lowering, e.g. the price of fruits and vegetables would really reduce the consumption of, e.g. soft drinks by children.

There is no doubt that these taxes cannot be a dramatic support to state budgets. But tobacco, alcohol and food related taxes together can bring considerable revenues. And they are taxes of “lesser evil”, since consumers can avoid them with their own healthier choices. And before all, they are a win–win situation for both state budgets and for effective improvement of public health, and consequently also for reducing health care and societal costs.

*Conflicts of interest*: None declared.
